# Novel environmental monitoring detector for discriminating fallout and airborne radioactivity

**DOI:** 10.1038/s41598-023-48730-0

**Published:** 2023-12-18

**Authors:** Philip Holm, Sakari Ihantola, Ville Bogdanoff, Kari Peräjärvi, Peter Dendooven, Olof Tengblad, Maarit Muikku

**Affiliations:** 1 Radiation and Nuclear Safety Authority, 01370 Vantaa, Finland; 2 Helsinki Institute of Physics, University of Helsinki, P.O. Box 64, 00014 Helsinki, Finland; 3 Instituto de Estructura de la Materia, CSIC, Serrano 113-Bis, 28006 Madrid, Spain; 4 Present Address: NeutronGate Oy, 11710 Riihimäki, Finland; 5 Present Address: Department of Physics, University of Jyvaskyla, P.O. Box 35, 40014 Jyvaskyla, Finland

**Keywords:** Environmental sciences, Engineering

## Abstract

Early warning networks are used for detecting abnormal radioactivity levels in the environment. State-of-the-art networks are equipped with both dose rate detectors and spectrometric stations. Current networks don’t automatically discriminate between radioactivity on the ground and in the air. A novel directional sensing gamma radiation detector utilizing a collimated phoswich scintillator was developed. The signals from the two scintillator materials are separated using a pulse shape discrimination. The separated signals are employed to determine the radioactivity concentrations on the ground and in the air assuming specific concentration distributions. Limitations related to imperfect directional sensing and dead time are discussed.

## Introduction

Early warning networks are an important part of nuclear accident preparedness. One of their tasks is timely detection of abnormal radioactivity levels in the environment. Networks were established^1^ in many European countries after the Chernobyl accident^2^, but e.g. in Finland the history of the external radiation monitoring network goes back to the 1960s^3^. Traditionally, dose rate detectors are used, but current state-of-the-art early warning networks are equipped with spectrometric stations to identify radionuclides. An effort to harmonize European early warning dosimetry networks by presenting recommendations and requirements for both dose rate detectors and spectrometers is presented in^1^. Requirements identified include sensitivity to changes in environmental dose rate, dose rate measurement range and readout frequency.

Present in-situ measurement stations have the inherent restriction of not being able to distinguish between airborne radioactivity and ground-deposited fallout. Operational intervention levels (OIL) are often expressed in terms of external dose rates, fallout radioactivity concentration or airborne radioactivity concentration^4,5^. E.g. in the report *Protective Measures in Early and Intermediate Phases of a Nuclear or Radiological Emergency*^4^, jointly published by the Nordic radiation protection and nuclear safety authorities, it is suggested that the intervention levels for adults taking iodine tablets are an external dose rate exceeding 100 µSv/h or an iodine concentration in inhaled air exceeding 10,000 Bq/m^3^ for two days. With present early warning networks using dose rate detectors or spectrometers, it is only possible to directly determine whether the external dose rate limit is exceeded.

The amount of radioactive nuclides in the air and on the ground can be determined by several methods. The most accurate approach is to collect soil and air samples that are then measured in a laboratory. Since this approach is both labor-intensive and slow, its usage is limited, especially in the early phase of a nuclear accident when results are required rapidly. Another approach is to perform in-situ measurements with collimated gamma-ray detectors that mainly record either the gamma-rays originating from the ground or the gamma-rays originating from the air. In earlier research this has been done by manually repeating the measurement with and without a gamma-ray shield^6,7^. Alternatively, the measurement station has two different gamma-ray spectrometers with different collimators^8,9^. A disadvantage of these solutions for airborne and fallout measurements is that they either require some manual work or double the number of gamma-ray detectors per measurement station.

This paper presents a prototype of a novel directional sensing gamma radiation detector utilizing a collimated phoswich scintillator. A phoswich scintillator is a combination of several scintillators. Different applications of phoswiches have been studied, such as atmospheric radioxenon monitoring^10^, hard X-ray astronomy^11^ and low-energy gamma-ray detection^12^. The in-situ method presented here does not require actively changing the measurement geometry nor performing multiple measurements. Because of this it could automatically perform some of the tasks of emergency responders and thus lessen their workload and accumulated radiation dose.

## Methods

### Detector assembly

**Figure 1:**
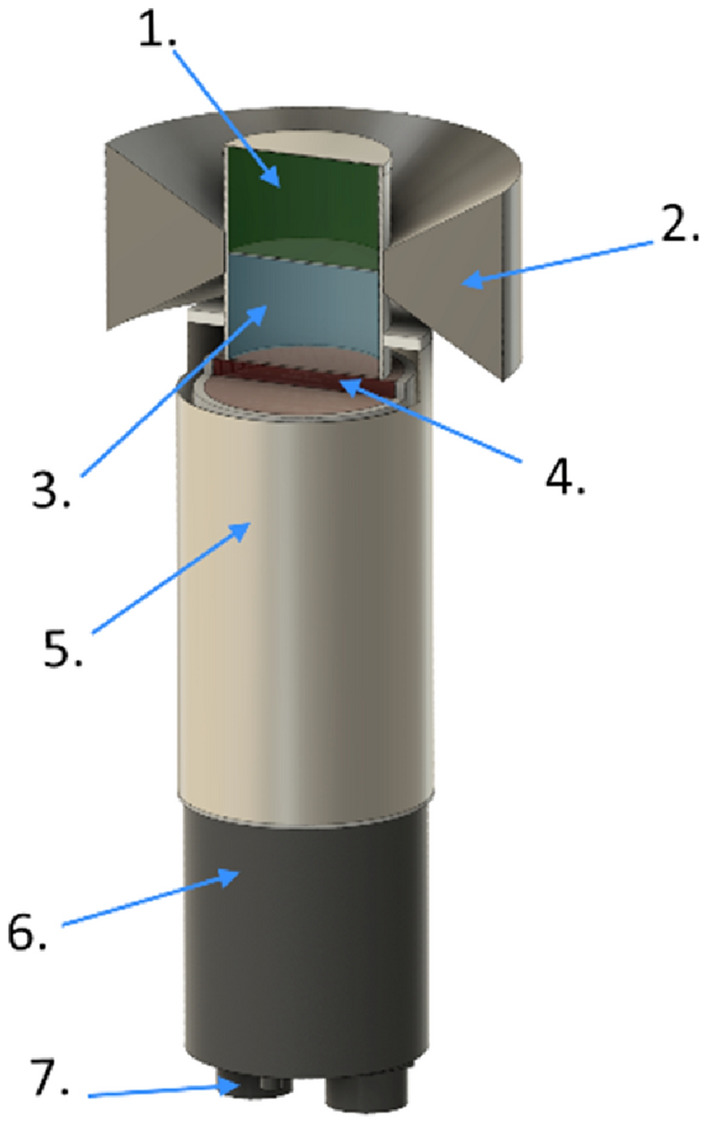
Cross-sectional view of the detector. 1—NaI(Tl) crystal, 2—Lead collimator, 3—CsI(Na) crystal, 4—Ø51 mm Hamamatsu R6231 PMT, 5—Magnetic shield, 6—Bridgeport Instruments usbBase, 7—USB port.

Figure 1 shows the prototype detector. The detector contains a phoswich scintillator package custom-made by Scionix Holland B.V. The package consists of vertically stacked thallium-doped sodium iodide NaI(Tl) and sodium-doped cesium iodide CsI(Na) scintillation crystals, each of 38 mm diameter and 25 mm length, optically coupled to a Hamamatsu R6231 photomultiplier tube (PMT). The diameter of the PMT is 51 mm and it is surrounded by a solid mu-metal magnetic shielding. The phoswich package is hermetically sealed with a light-tight 0.5 mm thick aluminum housing.

The choice of a NaI(Tl)/CsI(Na) phoswich was made after extensive laboratory testing of different combinations of scintillator crystals. This combination was selected for three reasons. Firstly, the light outputs of these scintillator materials feature distinct decay times so that the signals are easily discriminated. Secondly, these scintillator materials are transparent to each other’s scintillation light, enabling good light collection efficiency which is essential for good energy resolution. Thirdly, in NaI(Tl) and CsI(Na), the energy resolution is not highly degraded by the small losses in light collection efficiency, which are unavoidable in a phoswich design ^13,14^. The NaI(Tl)/CsI(Na) phoswich is also relatively inexpensive.

The detector is instrumented with a usbBase multichannel analyzer (MCA) made by Bridgeport Instruments that is plugged onto the 14-pin PMT socket. In addition to the MCA, the usbBase incorporates a high-voltage supply including the voltage divider. The MCA has a 12-bit ADC that operates with a speed of 40 megasamples per second. The usbBase is powered and controlled over USB.

The phoswich crystals are surrounded by a lead collimator that shields the top scintillation crystal from gamma radiation coming from the ground and vice versa shields the bottom scintillation crystal from gamma radiation coming from above the detector. The collimator has a cross-sectional shape of an isosceles trapezoid with a shorter base closer to the scintillators. The short and long base are 10 and 50 mm respectively. The inner and outer diameters are 43 and 100 mm respectively. The collimator design was optimized by Monte Carlo simulations made with the GEANT4 toolkit^15^.

### Pulse shape discrimination

The signals from the two scintillator materials can be separated by using pulse shape discrimination (PSD). Typically, the NaI(Tl) scintillator light pulse has a primary decay time of 0.23 µs^16^ at room temperature and CsI(Na) has a primary decay time of 0.63 µs^17^. Due to the very different decay times, the signals from these two scintillator materials are easy to separate.

To enable PSD, a custom firmware was loaded onto the usbBase MCA. With this firmware, the MCA records the scintillator events in list mode. Each entry in the list contains the time stamp, pulse integration over a long time window and pulse integration over a short time window for one scintillator event.

The long integration time was used to determine the energy of the gamma ray, and therefore, it was set long enough to capture most of the scintillation light emitted. After careful optimisation, an integration time of 4 μs was selected. Increasing the integration time further only slightly improved the CsI(Na) energy resolution and had no impact on the NaI(Tl) energy resolution. The slight improvement was considered not to justify the increased detector dead time that the longer integration time causes.

The short integration time was optimised for PSD. PSD performs best when the short integration time maximises the difference in integrated signals between the two scintillator materials. After testing various parameter values, the short integration time was set to 0.35 μs.

The parameter used for pulse shape discrimination was defined as

1 $$ PSD = \left( {I_{short} - C} \right)/\left( {I_{long} - C} \right) $$

where $I_{short}$ and $I_{long}$ are the pulse integration over the short and long time windows, respectively. The offset parameter $C$ does not have a clear physical meaning, but it was empirically noticed that if a constant value of 13 is subtracted from the pulse integration, the PSD parameter obtained does not depend on the pulse energy. Commonly used PSD models *PSD* = *I*_*short*_/*I*_*long*_ and *PSD* = 1 – *I*_*short*_/*I*_*long*_ were also tested but eventually not used, since the PSD parameters were energy depended^18,19^.

**Figure 2:**
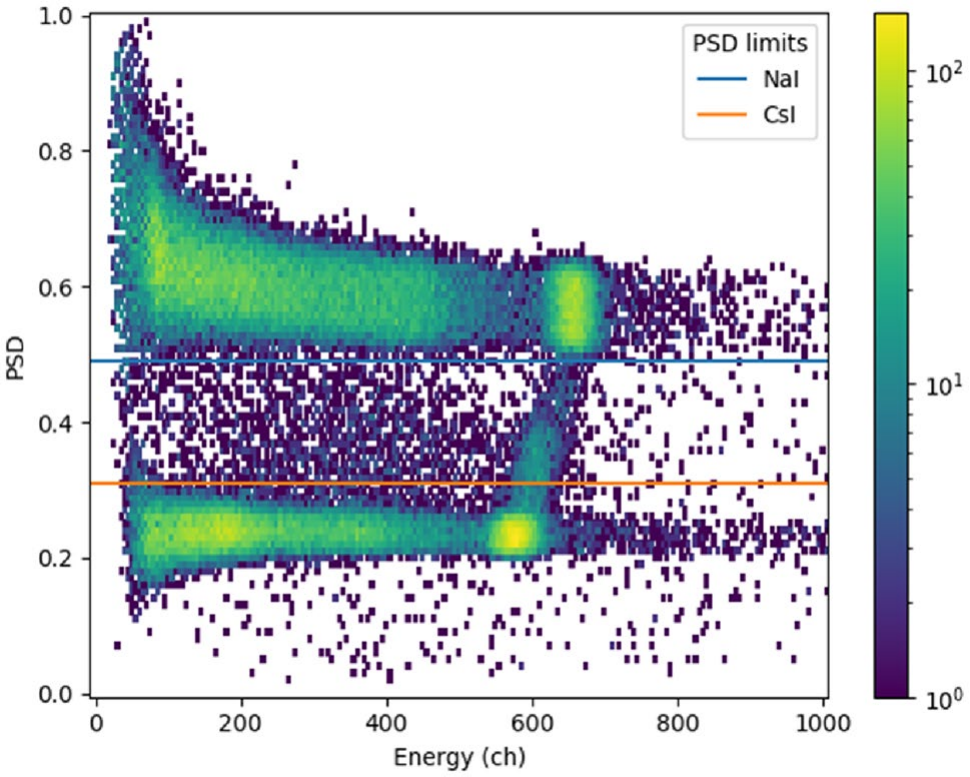
2D histogram of PSD versus energy of the gamma ray events recorded from a Cs-137 source at angle 0° (see Fig. 4 for angle). The horizontal axis represents the parameter used for energy reconstruction (signal amplitude integrated over 4 μs) and the vertical axis the parameter used for pulse-shape discrimination (calculated with Eq. 1). The blue horizontal line shows the minimum PSD value for NaI(Tl) events and the orange horizontal line the maximum PSD value for CsI(Na) events.

Figure 2 presents an example of a PSD plot for a measurement with a Cs-137 source that emits 662 keV gamma rays, placed on the centre axis above the NaI(Tl) scintillator (angle 0°; see Fig. 4 for angle). The two clusters at channels 580 and 660 are caused by full 662 keV deposition in the CsI and NaI scintillator, respectively (the detector was calibrated so that channel 662 corresponded to 662 keV for the NaI scintillator). The events left from the clusters are Compton scattered gamma rays with partial energy deposition in one detector. The diagonal line connecting the clusters is caused by complete 662 keV absorption shared between the two scintillator materials. Since the detector recorded only 1420 counts per second, random coincidences are hardly visible.

**Figure 3:**
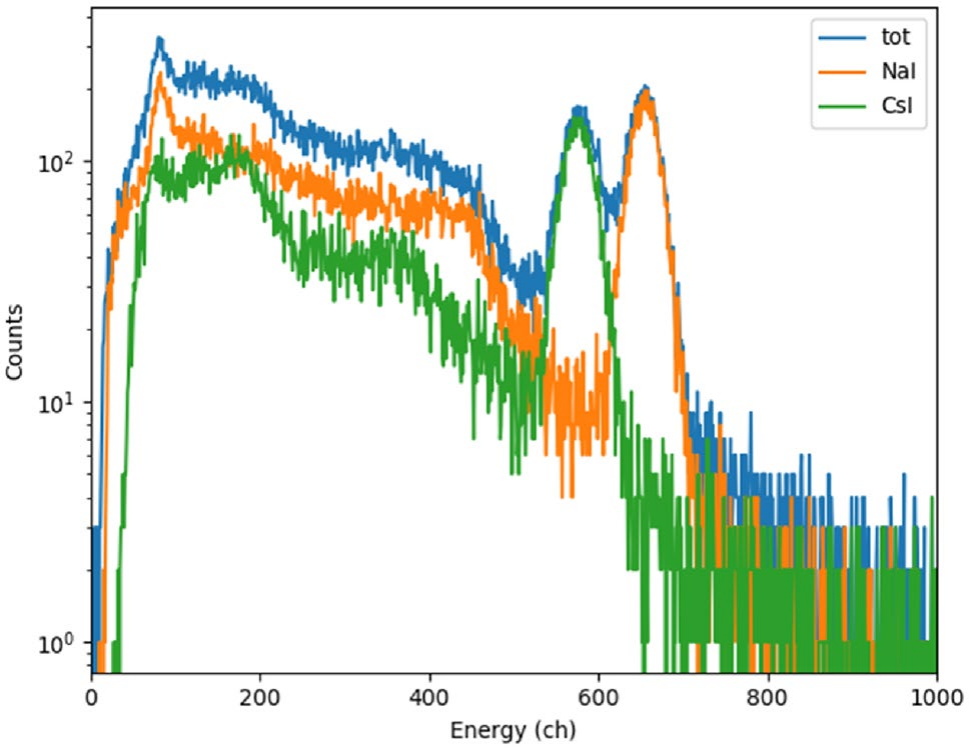
Undiscriminated (tot) and discriminated energy spectra of a Cs-137 source measured with the phoswich detector. The spectra are derived from the data presented in Fig. 2. The full absorption of 662 keV gamma rays from the Cs-137 source creates a peak at channel 660 in the NaI(Tl) scintillator spectrum and at channel 580 in the CsI(Na) scintillator. The different position of the peaks is mainly due to the difference in the light output of the two materials.

Events with the PSD parameter larger than 0.5 were considered to originate from the NaI(Tl) scintillator whereas events with the PSD parameter smaller than 0.3 were considered to originate from the CsI(Na) scintillator. The energy spectra obtained by applying pulse-shape cuts to the same data are presented in Fig. 3.

### Discriminating between airborne radioactivity and ground-deposited fallout

By PSD and gamma spectrum analysis, peak count rates can be extracted from the measurement data. Calibration factors that convert peak count rates to radioactivity concentrations need to be determined for specific fallout or airborne radioactivity geometries. Although the phoswich scintillators have a primary measurement direction and object (air and ground respectively), the collimator and the scintillators do not completely shield either scintillator from the opposing direction. The photopeak count rate S of the upper and lower scintillator in the phoswich can thus be described as

2 $$ S_{u} = \varepsilon_{V,u} A_{c} + \varepsilon_{A,u} A_{d} $$

3 $$ S_{l} = \varepsilon_{V,l} A_{c} + \varepsilon_{A,l} A_{d} $$

where ε_V_ is a calibration factor for the radioactivity in the air for a given cloud geometry and ε_A_ is the calibration factor for the radioactivity deposited on the ground. The indexes u and l represent the upper and lower scintillator. A_c_ represents the radioactivity concentration in the air (Bq/m^3^), and A_d_ represents the deposition density on the ground (Bq/m^2^). In the simplest case the concentrations are constant over the specified volume and area. In a more general case, the radioactivity concentrations A_c_ and A_d_ are linear coefficients of concentration distributions for which the corresponding calibration factors have been determined.

From Eqs. (2) and (3) we get

4 $$ A_{c} = S_{u} \frac{1}{{\varepsilon_{V,u} - \varepsilon_{V,l} \frac{{\varepsilon_{A,u} }}{{\varepsilon_{A,l} }}}} - S_{l} \frac{1}{{\frac{{\varepsilon_{A,l} }}{{\varepsilon_{A,u} }}\varepsilon_{V,u} - \varepsilon_{V,l} }} $$

5 $$ A_{d} = S_{l} \frac{1}{{\varepsilon_{A,l} - \frac{{\varepsilon_{V,l} }}{{\varepsilon_{V,u} }}\varepsilon_{A,u} }} - S_{u} \frac{1}{{\frac{{\varepsilon_{V,u} }}{{\varepsilon_{V,l} }}\varepsilon_{A,l} - \varepsilon_{A,u} }} $$

The calibration factors can be calculated at a gamma energy E for specific airborne and fallout geometries. Next, we calculate the calibration factors for an airborne activity in the shape of an infinite half sphere and an infinite fallout surface.

We assume that the detector efficiency does not depend on the azimuth angle due to symmetry. Let’s further assume that we know the absolute peak efficiency ε_p_(E, r_0_, θ) for a point source at a source-detector distance r_0_ as a function of the polar angle θ. This absolute peak efficiency can be determined experimentally as

6 $$ \varepsilon_{p} \left( {E,r_{0} ,\theta } \right) = \frac{{S\left( {E,r_{0} ,\theta } \right)}}{I\left( E \right)A} $$

where A is the activity of the point source, I(E) is the yield of photons with energy E and S(E, r_0_, θ) is the peak count rate of the scintillator.

For a detector at height h from the ground, the calibration factor can be calculated as a sum of two sets of integrals: one for the volume above the center of the detector and one for the volume below the center of the detector. This gives us

7 $$ \varepsilon_{V} = r_{0}^{2} e^{{\mu \left( E \right)r_{0} }} \left[ {\int_{0}^{2\pi } {\int_{0}^{\pi /2} {\mathop \int \nolimits_{0}^{\infty } } } \varepsilon_{p} \left( {E,r_{0} ,\theta } \right)e^{ - \mu \left( E \right)r} sin\left( \theta \right)drd\theta d\varphi + \int_{0}^{2\pi } {\int_{\pi /2}^{\pi } {\mathop \int \nolimits_{0}^{{h/cos\left( {\pi - \theta } \right)}} } } \varepsilon_{p} \left( {E,r_{0} ,\theta } \right)e^{ - \mu \left( E \right)r} sin\left( \theta \right)drd\theta d\varphi } \right] $$

where µ(E) is the attenuation constant in air and θ is the azimuthal angle.

The integral over θ can be estimated by measuring the absolute peak efficiency at distance r_0_ at m different polar angles θ_i_ from 0 to π (i.e. straight down to straight up) and interpolating ε_p_(E, r_0_, θ) linearly between the measured values at angles θ_i_. With θ_1_ = 0, θ_n+1_ = π/2 and θ_m_ = π we get

8 $$ \begin{aligned} \varepsilon_{V} & = 2\pi r_{0}^{2} e^{{\mu \left( E \right)r_{0} }} \mu \left( E \right)^{ - 1} \\ & \quad \left\{ {\mathop \sum \limits_{i = 1}^{i = n} \left[ {\left( {\varepsilon_{p} \left( {E,r_{0} ,\theta_{i} } \right)cos\left( {\theta_{i} } \right) - \varepsilon_{p} \left( {E,r_{0} ,\theta_{i + 1} } \right)cos\left( {\theta_{i + 1} } \right)} \right)} \right.} \right. \\ & \quad \left. { + \frac{{\varepsilon_{p} \left( {E,r_{0} ,\theta_{i + 1} } \right) - \varepsilon_{p} \left( {E,r_{0} ,\theta_{i} } \right)}}{{\theta_{i + 1} - \theta_{i} }}\left( {sin\left( {\theta_{i + 1} } \right) - sin\left( {\theta_{i} } \right)} \right)} \right] \\ & \quad \left. + \mathop \sum \limits_{i = n + 1}^{i = m - 1} \mathop \int \limits_{{\theta_{i} }}^{{\theta_{i + 1} }} \left( {1 - e^{{ - \mu \left( E \right)\frac{h}{{cos\left( {\pi - \theta } \right)}}}} } \right)\left( {\varepsilon_{p} \left( {E,r_{0} ,\theta_{i} } \right) + \frac{{\varepsilon_{p} \left( {E,r_{0} ,\theta_{i + 1} } \right) - \varepsilon_{p} \left( {E,r_{0} ,\theta_{i} } \right)}}{{\theta_{i + 1} - \theta_{i} }}\left( {\theta - \theta_{i} } \right)} \right)sin\left( \theta \right)d\theta \right \}\\ \end{aligned} $$

The remaining integral can be solved numerically.

The fallout calibration factor is calculated similarly. We assume that the detector is at height h from a flat circular surface

9 $$ \varepsilon_{A} = 2\pi r_{0}^{2} e^{{\mu \left( E \right)r_{0} }} \mathop \int \nolimits_{{t_{1} }}^{{t_{2} }} \varepsilon_{p} \left( {E,r_{0} ,t} \right)\frac{{e^{ - t} }}{t}dt $$

where

10 $$ t = \mu \left( E \right)\left( {k^{2} + h^{2} } \right)^{1/2} $$

and k is the distance from the center of the circle. By integrating by parts and letting the surface be infinite, we get

11 $$ \begin{aligned} \varepsilon_{A} & = 2\pi r_{0}^{2} e^{{\mu \left( E \right)r_{0} }} \mathop \sum \limits_{i = 1}^{i = n} \left[ {\left( {\varepsilon_{p} \left( {E,r_{0} ,t_{i} } \right) - \frac{{\varepsilon_{p} \left( {E,r_{0} ,t_{i + 1} } \right) - \varepsilon_{p} \left( {E,r_{0} ,t_{i} } \right)}}{{t_{i + 1} - t_{i} }}t_{i} } \right)\left( {E_{1} \left( {t_{i} } \right) - E_{1} \left( {t_{i + 1} } \right)} \right)} \right. \\ & \quad \left. { + \frac{{\varepsilon_{p} \left( {E,r_{0} ,t_{i + 1} } \right) - \varepsilon_{p} \left( {E,r_{0} ,t_{i} } \right)}}{{t_{i + 1} - t_{i} }}\left( {e^{{ - t_{i} }} - e^{{ - t_{i + 1} }} } \right)} \right] \\ \end{aligned} $$

where E_1_ refers to the exponential integral:

12 $$ E_{1} \left( x \right) = \mathop \int \nolimits_{x}^{\infty } \frac{{e^{ - t} }}{t}dt $$

## Results

### Angular response

**Figure 4:**
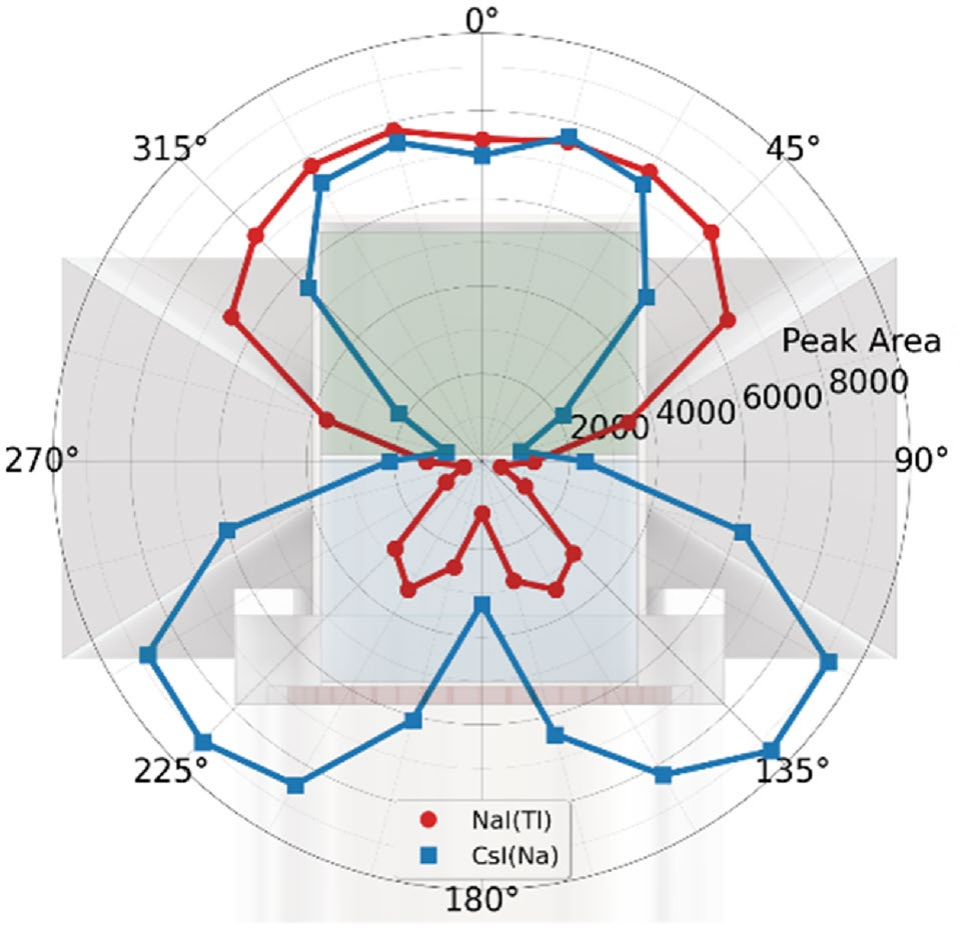
Polar plot of the phoswich response, measured with a 185 MBq Cs-137 source at 4 m distance and at different polar angles (θ). A cross section view of the detector is depicted in the background. The peak area values represent the number of counts in the 662 keV full energy peak obtained in 1-min measurements.

The angular response of the detector was measured with a 185 MBq Cs-137 source at a distance of 4 m. Cs-137 emits 662 keV photons with a yield of 0.851^20^ and has a significant role in releases during nuclear power plant accidents^21,22^. Measurements were performed at 15° intervals around the centre of rotation, i.e. the intersection point between the NaI(Tl) and the CsI(Na) crystals. Figure 4 presents the measured angular response of the 662 keV peak area. For operational use, a peak efficiency calibration should be done over the full energy region of interest.

The two scintillators have a clearly different angular response, especially in the downward direction. The smaller peak areas at angles 150°–210° are explained by the electronics below the scintillators (see Fig. 1).

### Calibration factors for fallout and airborne activity

Using the measured photopeak areas, Eqs. (6), (8) and (11), and a detector position of 1.5 m above ground level, we get the calibration factors presented in Table 1.

**Table 1:** Calibration factors for airborne activity and fallout at E = 662 keV and a detector position of 1.5 m above ground level.

	ε_V_ [m^3^]	ε_A_ [m^2^]
NaI(Tl) (top)	6.64 × 10^–3^ ± 4 × 10^–5^	3.68 × 10^–5^ ± 9 × 10^–7^
CsI(Na) (bottom)	4.78 × 10^–3^ ± 4 × 10^–5^	2.81 × 10^–4^ ± 3 × 10^–6^

The presented uncertainties are combined standard uncertainties calculated according to ^23^, taking into account only the statistical errors of the measured peak efficiencies.

For example, if we measure S_u_ = 100 s^−1^ and S_l_ = 200 s^−1^, using the values in Table 1 and Eqs. (4) and (5) we can calculate that the airborne radioactivity concentration is 12 kBq/m^3^ and the concentration deposited on the ground is 500 kBq/m^2^ (this example assumes a 100% photon yield).

## Discussion

According to IAEA safety standards, operational intervention levels (OILs) for initiating different parts of emergency plans should be established^5^. The Nordic radiation protection and nuclear safety authorities have published joint guidelines and recommendations for protective measures in emergencies^4^. In STUK et al.^4^, the OIL for sheltering the population indoors due to airborne intense gamma and beta emitters such as Cs-137 is 10 kBq/m^3^. Similarly, STUK et al.^4^ determines the OIL for continuing sheltering indoors because of deposited intense gamma and beta emitters as 10 MBq/m^2^. This level of contamination is defined as “extreme” in^4^. According to^4^ with Cs-137, this fallout activity concentration corresponds to an external dose rate of 25 µSv/h.

A detector should be optimized for its planned use. There are several factors limiting the use of any spectrometer in a given situation, such as deadtime and energy resolution. These factors have to be taken into account also in the design of, and operational plans for, the phoswich detector.

The deadtime of the present detector without the collimator was measured at an angle of 90° (see Fig. 4) with different dose rates caused by a Co-60 source. With an ambient dose equivalent rate of 22 µSv/h, the dead time was around 20%. The collimator and energy spectrum have an effect on the deadtime, but the measurement result hints at a detector limitation because of unreasonable deadtime close to the OIL fallout activity defined in^4^. There is a trade-off between detection efficiency and deadtime at high dose rates.

**Figure 5:**
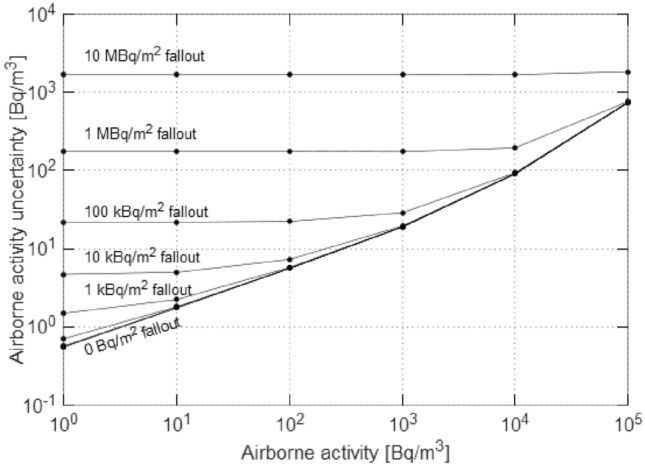
Combined standard uncertainty of the calculated airborne Cs-137 activity vs. airborne activity, for different fallout Cs-137 concentrations. The uncertainties are calculated according to ^23^, assuming a 10-min measurement, using the calibration factors and respective uncertainties in Table 1, and using the square-root of the peak area as an estimate for the peak area uncertainty.

The limitations of discriminating fallout from airborne radioactivity with the phoswich detector can be studied. Figure 5 presents the effect of increasing fallout concentrations on the uncertainty of the calculated airborne activity concentration. With a 10 MBq/m^2^ Cs-137 fallout and 10 kBq/m^3^ Cs-137 air concentration, the relative uncertainty of the estimated air concentration is around 18%. Because of stronger attenuation, the discrimination capabilities should be better for lower gamma energies.

One factor limiting the use of a stationary in-situ gamma spectrometer in a fallout situation is contamination of the detector surface. This is an issue also for the detector presented here and limits its discriminating capabilities. The effect of contamination can be reduced by covering the in-situ detector with a plastic cover that can be changed. The possible contamination could also be measured and discriminated from the fallout and airborne radioactivity by attaching a third scintillator to the phoswich package^24^.

It is worth noting that the uncertainty related to the radioactivity distribution introduces significant uncertainties in the calibration factors. This is also a general problem of in-situ measurements of radioactivity in the air or on the ground. In reference^25^, methods for assessing the representativeness of a measurement site are presented. In reference^7^, calibration factors correcting for the differences between a semi-infinite cloud and slabs of finite thickness are calculated. Reference^7^ states that the semi-infinite approximation introduces an error of 20% compared to a 180 m thick slab source. Similar geometry calculations could be done to study the effect on the discriminating capabilities of the phoswich detector. The calibration factors of the detector can be calculated for different geometries to improve the accuracy and precision of the activity estimates and thus improve the response in emergency management.

## Conclusion

The phoswich detector presented here demonstrates that it is possible to automatically discriminate fallout and airborne radioactivity using only one detector and one in-situ measurement geometry. If needed, the design and performance can be further optimized to specific operational tasks of the early warning network. Using the phoswich detector, the network can automatically provide information on the physical quantities used in the operational intervention levels.

## Data Availability

The datasets used and/or analyzed during the current study can be made available under a collaboration agreement with The Radiation and Nuclear Safety Authority of Finland. Please contact the corresponding author for more information.
